# Manic episode following psilocybin use in a man with bipolar II disorder: a case report

**DOI:** 10.3389/fpsyt.2023.1221131

**Published:** 2023-09-22

**Authors:** Haniya J. Halim, Bradley G. Burk, Rachel E. Fargason, Badari Birur

**Affiliations:** ^1^PGY3 Psychiatry Resident, Department of Psychiatry and Behavioral Neurobiology, University of Alabama at Birmingham, Birmingham, AL, United States; ^2^Department of Pharmacy, University of Alabama at Birmingham, Birmingham, AL, United States; ^3^Department of Psychiatry and Behavioral Neurobiology, University of Alabama at Birmingham, Birmingham, AL, United States; ^4^Department of Psychiatry and Behavioral Neurobiology, University of Alabama at Birmingham, Birmingham, AL, United States

**Keywords:** substance-induced mania, psilocybin, bipolar disorder, magic mushrooms, psychedelic

## Abstract

There has been an increase in research on the topic of psychedelic substances and their effects as treatment options in neuropsychiatric conditions. Psilocybin is a psychedelic drug that has recently garnered increased interest as an effective treatment modality for treatment-resistant depression, depression associated with terminal conditions, certain substance use disorders, and obsessive-compulsive disorder. However, sparse data exist as to the effects that psilocybin might have on patients at risk for mania, in large part secondary to the exclusion of this patient population from studies due to the concern for inducing mania or worsening illness course. We describe a case of a 21-year-old male with a recent diagnosis of bipolar II disorder who developed a manic episode following the ingestion of psilocybin in the form of hallucinogenic mushrooms. Given the incidence of depression in those with bipolar disorder, impulsivity, and a tendency to abuse substances associated with the illness, further research is needed into the risks of psilocybin and other psychedelic use in those with bipolar disorder.

## 1. Introduction

Psilocybin is a psychedelic agent principally found in fungi, particularly mushrooms from the genus *Psilocybe* (colloquially known as “magic mushrooms”). It has been used for centuries in various religious and spiritual ceremonies and, more recently, has been studied as a therapeutic option for psychiatric conditions (1). Psilocybin is a prodrug dephosphorylated into the active compound psilocin, which binds with high affinity to the serotonin 2A receptor (5-HT_2A_) and lower affinity to other serotonergic receptors (2). Similarly, to lysergic acid diethylamide (LSD), the potent agonistic effects of psilocybin at the 5-HT_2A_ receptor have been shown to induce hallucinatory experiences (3). As evidenced by various studies, activation of 5-HT2A receptors likely increases the release of dopamine from the mesocortical and nigrostriatal systems (4, 5) with resulting psychomimetic effects. In a review of the literature (PubMed and Google Scholar) looking at case reports involving adverse psychiatric effects following psychedelics, 18 cases were found involving the incidence of mania, five of which involved psilocybin (6). Psilocybin has been found to be effective as a treatment modality for treatment-resistant depression (7), depression associated with terminal illnesses (8, 9), and obsessive-compulsive disorder (10), to name a few. However, patients with bipolar disorder have been excluded from many of these studies due to the potential risk of inducing substance-induced mania with a full serotonin agonizing agent (6, 9). Therefore, little is known about the effects of psilocybin in the bipolar population, for which delay in diagnosis can lag for years following a major depression diagnosis due to the natural progression of the illness. A web-based survey containing observational data of patients with self-reported bipolar disorder who had used psilocybin to achieve a full psychedelic effect reported that a third of respondents experienced an adverse effect such as new or worsening manic symptoms (11). Clinicians should be aware that the risk of adverse outcomes increases as the use of psilocybin as a treatment for depression rises, and as the treatment settings move from heavily screened trials to less supervised clinical sites. In this report, we present a case of a patient with bipolar II disorder who had his first manic episode following ingestion of large amounts of psilocybin in the form of hallucinogenic or psilocybin-containing mushrooms. This report aims to add to the existing limited literature on psilocybin-induced mania as well as serves as a cautionary tale.

## 2. Case presentation

The patient is a 21-year-old Caucasian male with a past psychiatric history of recently diagnosed bipolar disorder and attention deficit hyperactivity disorder who presented to the emergency department accompanied by his mother with concern for mania and psychosis. Psychiatric and medication history was obtained from both the patient and his mother. He had started seeing a private psychiatrist 4 months prior, during which time he was prescribed fluoxetine and lisdexamfetamine for depression and attention deficit hyperactive disorder. The private psychiatrist had diagnosed the patient with bipolar II disorder, but per patient and his family, he had never been treated with a mood stabilizer or antipsychotic medication. He was frequently non-adherent to his medications and discontinued them completely 2 months prior to this episode. His parents also described intermittent elevations in mood, which appeared consistent with hypomania, occurring prior to the start of the antidepressant and stimulant medications. They were described as periods occurring every 4 to 5 months where the patient did not sleep for 1–2 days, spent more money than usual, and had increased goal-directed activity. His parents attested that these episodes occurred without the influence of any substances. They were able to manage his episodes at home, and he had never behaved impulsively to the point of risking his life or requiring hospitalization. Their family history was positive for completed suicide in his father and diagnoses of bipolar disorder in his father and several members of his mother’s family.

**Figure 1:**
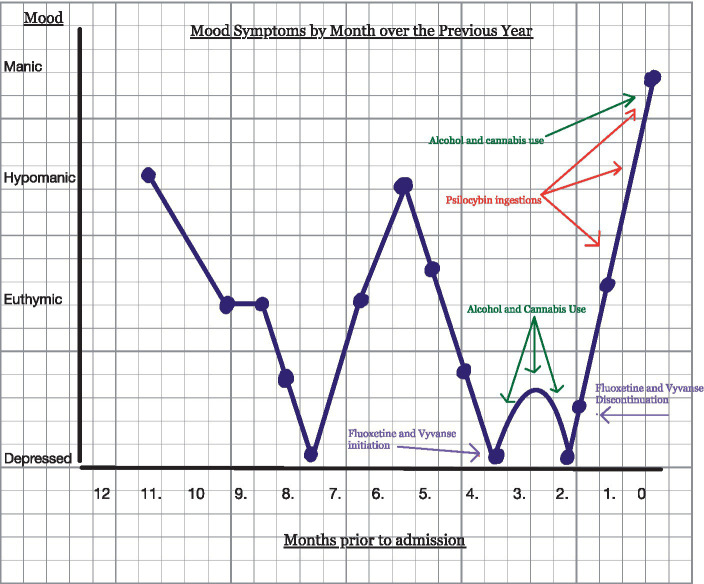
Timeline events of the patient’s mood map over the previous year.

Figure 1 depicts events in a mood map/ timeline format. The patient used psilocybin for the first time with friends several times in the weeks prior to admission and only experienced the immediate euphoric effects associated with the substance. The effects were said to wear off within several hours, returning him to his normal euthymic state. He then visited the beach with friends, where, along with alcohol and cannabis, he ingested cannabis four to five times a week. He was not able to remember exactly how much psilocybin was consumed. Two days following his last use, he developed symptoms consistent with mania, including elevated mood, increased energy, grandiosity, and increased risk-taking behaviors. When driving home from the beach trip, he increased the speed of the car to >135 mph on the freeway “to see how fast he could go.” He collided with another vehicle but was not injured. He then began to run on the shoulder of the freeway to “test his abilities.” He removed most of his clothing and shoes while running “because they slowed him down.” He was grazed by a passing truck but again was not injured. The truck driver summoned the police to the scene. The patient then ran away from the police but was eventually contained. After being released on bail, he spent the night in jail and walked alongside the road for 1.5 days without rest to eat or drink. The police picked him up again while sitting in the middle of high-speed traffic on the highway. His family was called to take him to the emergency department. On admission, the patient displayed elevated energy levels and mood, psychomotor agitation in the form of pacing, racing thoughts, flight of ideas, fast speech, and expanded affect. His vital signs were within normal limits, and initial labs obtained were normal except for elevated creatine kinase (1,600 units/L) and urine drug screen positive for cannabis with an alcohol level of less than 10 mmol/L. Imaging included computed tomography (CT) of the head without contrast, which showed no acute intracranial processes. The physical exam was significant for open sores on his feet, obtained while running barefoot for many miles. He remembered vividly the events leading to admission but displayed poor insight and did not demonstrate any remorse for his actions.

Our diagnosis for this patient was substance-induced bipolar disorder, manic type. Once admitted, he was treated with divalproex sodium delayed-release and risperidone. Due to increased psychomotor agitation, lorazepam was started and eventually tapered. By discharge, the patient displayed improved insight and a decrease in energy, delusional thinking, and expansive mood. A three-week hospital stay was required to stabilize his mood. He was discharged home to the family on divalproex sodium delayed-release tabs 500 mg twice daily, risperidone 4 mg daily with outpatient follow-up scheduled, and substance use counseling arranged. As the psilocybin use may have preempted his manic episode, he was cautioned against further use of hallucinogens and other substances. Six months post-discharge, a follow-up phone call was made to the patient. He stated he was faring well, was compliant with medications, and seeing his outpatient psychiatrist regularly. He denied any further manic or depressive episodes since discharge.

## 3. Discussion

We present a case of a patient with a previous history of hypomanic and depressive episodes and a first-degree family history of bipolar disorder. In the context of multiple episodes of psilocybin ingestion along with other illicit substances, and without the use of antidepressants or mood stabilizers, this patient developed a first-time full manic episode. DSM-V defines substance-induced bipolar disorder as a prominent and persistent disturbance in the mood with manic predominance that is characterized by an elevated, expansive, or irritable mood that developed during or soon after substance intoxication or withdrawal. From collateral obtained from the parents, it was determined that although the patient had a recent trial of fluoxetine for depression and lisdexamfetamine for ADHD, his last ingestion of these medicines was six weeks prior to the development of any of the manic symptoms leading to admission. This time-lapse reduced the possibility that he was experiencing a medication-induced mania from an antidepressant or a stimulant.

Strong serotonin agonism is postulated to increase dopamine transmission within the mesocortical and nigrostriatal pathways (4, 5). Similar to other pro-serotonin agents, psilocybin, with its agonist action on 5-HT_2A,_ can possibly flip a euthymic individual with bipolar propensities into a manic state. This may be akin to the effects of an antidepressant with serotonergic affinity inducing mania in an individual with a diagnosis or predisposition to bipolar disorder (12). A similar example is seen in a case report where a 21-year-old female without a history of bipolar disorder is noted to have experienced an episode of mania preceded by psilocybin and cannabis use (13). This patient also had a prolonged history of cannabis use as well as recent treatment of depression with fluoxetine. In contrast to this report, the patient in our case was recently diagnosed with bipolar II disorder due to a history of depressive and hypomanic episodes. A case review by Gard et al. (6) details 17 case reports of manic episodes following hallucinogen use in various forms, including psilocybin, LSD (lysergic acid diethylamide), DMT (N, N-Dimethyltryptamine), and ayahuasca (a psychotropic, hallucinogenic beverage composed of a mixture of DMT and a monoamine oxidase inhibitor (MAOI) (6). Two of the 17 cases shared similarities with ours in that they described individuals with a diagnosis of bipolar disorder who experienced psychiatric morbidity following the use of psychedelic substances. The first case reported a psychiatrist with bipolar 1 disorder who self-medicated with the hallucinogen DMT paired with the MAOI phenelzine, resulting in an episode of mania and psychosis (2). Similar to psilocybin, DMT also has agonist activity at 5HT_2A_ and 5HT_1A_ receptor sites. The second case describes an Argentinian male with bipolar II disorder who used the hallucinogen ayahuasca for recreational use and developed his first manic episode. Ayahuasca is considered to have antidepressant properties based on its inhibitory action on serotonin reuptake and agonism of the serotonin 1A receptor, as well as reversible inhibition of the MAO-A enzyme (12). Four cases in the review by Gard et al. (6) consisted of individuals with a family history of bipolar disorder but no personal history. These data highlight the risk of ingesting hallucinogens in those with bipolar disorder or a predisposition to bipolar disorder, such as positive family history. Our case may be the first known report of the possible induction of mania in an individual with bipolar II disorder through psilocybin use.

Several studies have been published highlighting the use of psilocybin use for the treatment of obsessive-compulsive disorder, treatment-resistant depression, and some substance use disorders (10, 14–16). The favorable results of these studies indicate that psilocybin may soon be an FDA-approved and evidence-based treatment for depression, anxiety, substance use disorders, OCD, and even PTSD. Many patients with bipolar disorder share these comorbid diagnoses and run the risk of seeking psilocybin and having an adverse outcome due to the induction of mania. In the psilocybin studies cited above, thorough screening was conducted and patients with bipolar disorder or family history of BPAD were excluded. However, as the medicalization of psilocybin proceeds, administration for the treatment of depression will likely move from clinical trials to less supervised settings, inevitably with a decline in the quality of pre-treatment screening. This advancement will leave more patients at risk for mania induction, and therefore it is vital for clinicians to maintain a high level of scrutiny when administering psilocybin to patients, especially those who are younger ages. Looking at the natural course of the development of bipolar disorder, adolescents and young adults are more likely to develop depression prior to the first activated (hypomanic/manic) episode (17). One study found that the average latency period between an adolescent/young adult’s depressive episode and the first activated episode was 4.9 years (17). This delay in the progression of symptoms allows for an opportune time for treatment with various antidepressants including psilocybin, which may lead to adverse outcomes mentioned above.

In the web-based survey (11) observing patients with self-reported bipolar disorder using psilocybin, it was noted to be expected that those with bipolar I disorder would be more likely to report adverse effects such as mania with psilocybin use. In their survey findings, however, it was found that there was no difference between adverse or unwanted effects between bipolar I and bipolar II patients (11). Our case, along with a case involving ayahuasca in Gard et al.’s (6) review, indicates it is possible for a serotonin agonist to trigger a first manic episode in a patient with bipolar II disorder. Additionally, it has been demonstrated that substance use tends to start around the same time as the first major mood episode in the lifetime of BPAD patients (17). As these patients have a greater propensity to engage in high-risk behaviors, they are more likely to experience the effects of potentially activating substances such as hallucinogens compared to patients without mood disorders. Providers should be aware of these risks and warn patients who may be prone to illicit or legitimate use involving hallucinogens.

Conclusions drawn from this report are limited due to the subjectivity of historical information obtained from the patient’s family, lack of information on dosing or purity of psilocybin agent consumed, concurrent marijuana and alcohol use that confounds interpretation, and the relatively recent previous trial with antidepressants. Future areas of research may assess whether pre-treating patients with bipolar disorder with an antipsychotic or mood stabilizer prior to using psilocybin decreases the risk of inducing mania or if with meticulous dosing and monitoring, patients with bipolar II disorder in a depressive phase can achieve some benefit with the substance without adverse effects.

## 4. Conclusion

We describe a patient with a history of bipolar II disorder who used significant amounts of psilocybin in the form of magic mushrooms and experienced a manic episode. He required nearly a three-week hospitalization and treatment with a mood stabilizer and antipsychotic before his symptoms abated. He had had no prior knowledge of the risk of inducing a manic episode from magic mushrooms with his history. This report highlights the potential for a serious adverse outcome from the recreational use of psilocybin in this at-risk population, likely due to its agonist action on the 5HT_2A_ receptor. As the substance grows in popularity as a treatment for resistant depression and anxiety, clinicians must be aware of the risk and warn their patients accordingly.

## Data availability statement

The original contributions presented in the study are included in the article/supplementary material, further inquiries can be directed to the corresponding author.

## Ethics statement

Written informed consent for the publication of this case report was obtained from the patient.

## Author contributions

HH and BrB were involved in the assessment and medication management of the patient. HH wrote the first draft of the manuscript. BaB, BrB, and RF contributed to the preparation of the manuscript and have approved the final version of the manuscript.

## Conflict of interest

The authors declare that the research was conducted in the absence of any commercial or financial relationships that could be considered a potential conflict of interest.

## Publisher’s note

All claims expressed in this article are solely those of the authors and do not necessarily represent those of their affiliated organizations, or those of the publisher, the editors and the reviewers. Any product that may be evaluated in this article, or claim that may be made by its manufacturer, is not guaranteed or endorsed by the publisher.
